# Inequalities in women’s utilization of postnatal care services in Bangladesh from 2004 to 2017

**DOI:** 10.1038/s41598-022-06672-z

**Published:** 2022-02-17

**Authors:** Samia Aziz, Abdul Basit, Saima Sultana, Caroline S. E. Homer, Joshua P. Vogel

**Affiliations:** 1 Maternal, Child and Adolescent Health Program, Burnet Institute, Melbourne, Australia; 2 School of Population and Global Health, University of Melbourne, Melbourne, Australia; 3 Institute of Statistical Research and Training, University of Dhaka, Dhaka, Bangladesh; 4 Projahnmo Research Foundation, Dhaka, Bangladesh

**Keywords:** Health care, Risk factors

## Abstract

Postnatal care (PNC) is an essential component of maternity care. Appropriate and timely care immediately after childbirth can save lives and help to prevent or treat comorbidities resulting from pregnancy and childbirth. Despite its importance, PNC coverage is still low in Bangladesh. The aim of this study was to analyse the trends, inequalities, and factors associated with PNC for mothers in Bangladesh. Data from the last five Bangladesh Demographic and Health Surveys (BDHS) were used. Descriptive statistics were used to report PNC outcome rates and trends across six inequality indicators. Modified Poisson regression analyses were used to identify factors associated with PNC use in the most recent BDHS. A total of 21,240 women were included for the analysis. The rate of PNC by ‘medically trained provider’ within 2 days of birth increased between 2004 and 2017, from 16 to 52%. There were wide inequalities across socio-demographic factors. The regression analyses found women giving birth at home, women from the poorest wealth quintile and women receiving no antenatal care (ANC) were least likely to receive PNC. The findings emphasize the need to improve public health programs supporting women who have the least access to PNC. The identified inequalities can inform policy formulation to ensure more equitable provision of PNC to women in Bangladesh.

## Introduction

In the past 25 years there have been remarkable achievements in preventing maternal mortality, with a 37% reduction in maternal deaths between 2000 to 2017^1^. Despite such progress, an estimated 295,000 women globally and 5100 women in Bangladesh died due to pregnancy and related complications in 2017^1^. Approximately 50% of all maternal deaths occur within the first 24 h after childbirth and more than two-thirds occur within a week of childbirth^2^. An estimated 60% of all maternal deaths could be prevented if women were to receive appropriate postnatal care^3^.

The postnatal period begins immediately after childbirth and extends up to 42 days after birth—it represents a critical time period for maternal and neonatal survival^4^. WHO recommends that all mothers and newborns should receive postnatal care (PNC) from a skilled health provider within the first 24 h of birth irrespective of the place of birth, and they should also receive at least three additional postnatal check-ups within 42 days of birth^2^. Routine postnatal visits for a mother typically involve early detection, treatment and prevention of complications including postpartum hemorrhage, eclampsia and puerperal sepsis, assessment of breastfeeding progress, and support and advice on emotional wellbeing^2,4^. Despite these recommendations, globally about 40% women do not receive postnatal visits^5^ and only less than half of women receive care within 24 h of birth^6^.

Health inequalities are broadly defined as the unfair and preventable differences in health of population or across different groups of a population^7^. Inequalities in access to maternity health services for different socio-economic groups, both within and across countries, are well-documented^8,9^. Addressing these inequalities is likely to rectify the high rates of preventable maternal and perinatal morbidity and mortality in LMIC’s such as Bangladesh. For example, the risk of maternal mortality has been shown to be highest amongst women in the poorest wealth quintile and in rural areas^10^. A 2015 systematic review showed that in LMICs, utilization of postnatal care varied widely according to women’s socio-economic status, geographical location and maternal and partner’s education^11^. These inequalities pose a huge challenge in achieving the 2030 targets for the Sustainable Development Goals as well as progressing towards universal health coverage (UHC)^12^.

Bangladesh, located in southeast Asia, is one of the most densely populated countries in the world (1252 people per km^2^) with a total population of more than 160 million (2020)^13,14^. The population is relatively young—40% of people are aged between 25 and 54 years, and 38.2% live in urban areas^15^. The country has observed a gradual but substantial decline in maternal mortality ratio (MMR) from 319 per 100,000 live births in 2005 to 173 per 100,000 in 2017, an average annual rate of reduction of 4.7%^10^. However, to reach the UN Sustainable Development Goal maternal mortality target of less than 70 deaths per 100,000 live births by 2030^12^, MMR reduction needs to accelerate in Bangladesh.

Four postnatal check-ups are recommended for all women in Bangladesh^16^, and the Government has set a target to achieve 80% postnatal care coverage within 48 h from a ‘medically trained provider’ by 2025 and 100% by 2030^17^. While several studies have previously reported the low coverage of postnatal care in Bangladesh^18–21^, there is very little research that has explored factors associated with low postnatal care access and usage^18,22–26^. Studies conducted by Pulok et al. and Mahabub et al. examined inequality in overall maternal health care utilization across geographical location, wealth index and expenditure of services related indicators^27,28^. In addition, Hajizadeh et al.^29^ explored trends in utilization of antenatal and delivery care coverage across different socio-demographic groups. However, previous studies have not explored the relationship between postnatal care and socio-demographic indicators in Bangladesh. Therefore, this study quantifies the extent of inequalities in PNC utilization across six key socio-demographic indicators, which are known to influence maternal service utilization in different studies in Bangladesh^23,27,28,30,31^. Secondly, it also tries to identify the predictors of low PNC utilization using the most recent household survey data, which is imperative to address the further decline in MMR. Identifying drivers of low PNC utilization can help policymakers to design more effective intervention strategies crucial to improve maternal health and survival rates of neonates in Bangladesh. Thus, the aim of this study was to analyse the trends and inequalities in women’s utilization of PNC in Bangladesh from 2004 to 2017 using national representative surveillance data. The objectives were to estimate the trends in PNC coverage in Bangladesh between 2004 to 2017, assess these trends by key measures of inequality (place of birth, residence, geographical division, religion, maternal education, and wealth index), and identify factors associated with low PNC coverage.

## Results

**Figure 1:**
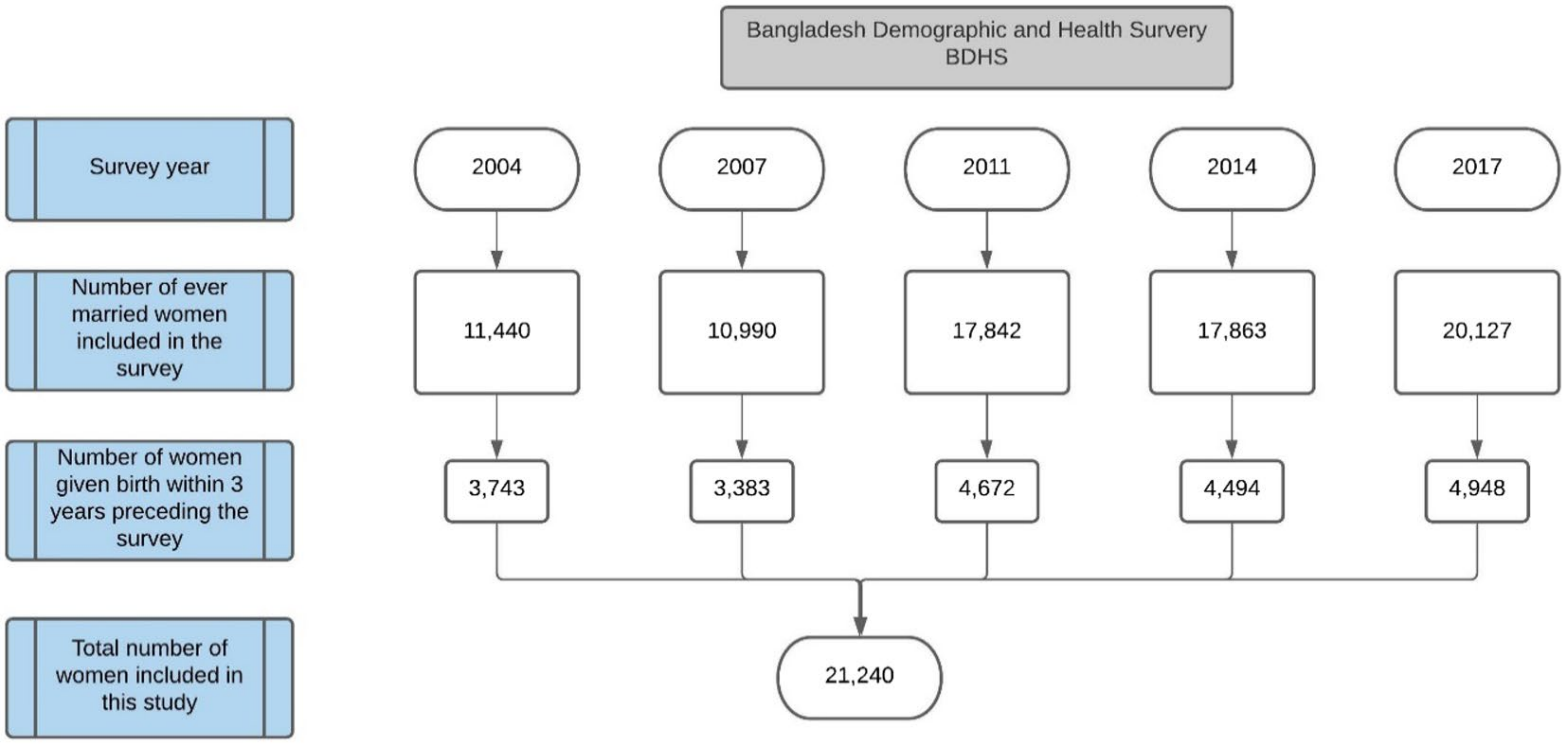
Analysis population. Ever married women who had given birth in the three years preceding the survey were included from five representative household surveys in Bangladesh (2004 to 2017).

Table 1 reports the characteristics of women giving birth in the three years preceding each of the five surveys. In total 21,240 women were included in the analysis (Fig. 1). The proportion of women giving birth in a health facility increased over time—from 13.9% in 2004 to 50.3% in 2017. The percentage of women with no formal education decreased from 33.5% in 2004 to 6.2% in 2017. The proportion of women across the place of residence, geographical divisions and wealth quintiles did not change meaningfully between 2004 and 2017.

**Table 1:** Percentage distribution of socio-demographic and maternal characteristics of the women given birth three years preceding the survey in 2004, 2007, 2011, 2014 and 2017.

	Survey year				
Socio-demographic characteristics	2004	2007	2011	2014	2017
	N = 3743	N = 3383	N = 4672	N = 4494	N = 4948
	n (%)	n (%)	n (%)	n(%)	n (%)
Place of birth					
Institutional birth	521(13.9)	684(20.2)	1471(31.5)	1794(39.9)	2492(50.4)
Home birth	3231(85.8)	2689(79.5)	3190(68.3)	2693(59.9)	2442(49.3)
Others	9(0.2)	10(0.3)	11(0.2)	7(0.2)	14(0.3)
Residence					
Urban	1134(30.3)	1205(35.6)	1481(31.7)	1451(32.3)	1697(34.3)
Rural	2609(69.7)	2178(64.4)	3191(68.3)	3043(67.7)	3251(65.7)
Divisions^1^					
Barisal	408(10.9)	422(12.5)	526(11.3)	532(11.8)	526(10.6)
Chittagong	807(21.6)	699(20.7)	942(20.2)	862(19.2)	826(16.7)
Dhaka	819(21.9)	728(21.5)	755(16.2)	795(17.7)	736(14.9)
Khulna	503(13.4)	400(11.8)	552(11.8)	531(11.8)	518(10.5)
Rajshahi	709(18.9)	570(16.8)	593(12.7)	546(12.1)	517(10.4)
Rangpur^a^	/	/	593(12.7)	550(12.2)	550(11.1)
Sylhet	497(13.3)	564(16.7)	711(15.2)	678(15.1)	677(13.7)
Mymensingh^b^	/	/	/	/	598(12.1)
Religion					
Islam	3420(91.4)	3073(90.8)	4197(89.8)	4134(92.0)	4533(91.6)
Hindu and others	323(8.6)	310(9.2)	475(10.2)	360(8.0)	415(8.4)
Mother’s education					
No education	1252(33.4)	802(23.7)	774(16.6)	607(13.5)	307(6.2)
Primary	1157(30.9)	1029(30.4)	1369(29.3)	1235(27.5)	1377(27.8)
Secondary	1086(29.0)	1264(37.4)	2118(45.3)	2130(47.4)	2368(47.9)
Higher	248(6.6)	287(8.5)	411(8.8)	522(11.6)	896(18.1)
Wealth index					
Poorest	843(22.5)	637(18.8)	1001(21.4)	940(20.9)	1066(21.5)
Poor	698(18.6)	689(20.4)	877(18.8)	855(19.0)	1007(20.3)
Middle	724(19.3)	616(18.2)	893(19.1)	860(19.1)	892(18.0)
Richer	661(17.7)	647(19.1)	948(20.3)	946(21.0)	972(19.6)
Richest	817(21.8)	794(23.5)	953(20.4)	893(19.9)	1011(20.4)
Maternal characteristics					
Mother’s age at last birth					
< 20	1168(31.2)	1012(29.9)	1405(30.1)	1406(31.3)	1400(28.3)
20–34	2365(63.2)	2185(64.6)	3076(65.8)	2904(64.6)	3357(67.8)
35–49	210(5.6)	186(5.5)	191(4.1)	184(4.1)	191(3.9)
Sex of child					
Female	1859(49.5)	1674(49.5)	2307(49.4)	2173(48.3)	2354(47.6)
Male	1884(50.3)	1709(50.5)	2365(50.6)	2321(51.6)	2594(52.4)
Child is twin					
singleton	3707(99.0)	3356(99.2)	4626(99.0)	4464(99.3)	4896(98.9)
Multiple	36(1.0)	27(0.8)	46(1.0)	30(0.7)	52(1.0)
Birth order					
1	1135(30.3)	1140(33.7)	1721(36.8)	1826(40.6)	1893(38.2)
2–3	1593(42.6)	1490(44.0)	2146(45.9)	2042(45.4)	2454(49.6)
4–5	654(17.5)	525(15.5)	612(13.1)	476(10.6)	196(10.0)
6 and above	361(9.6)	228(6.8)	193(4.1)	160(3.3)	105(2.1)
Woman experienced a miscarriage, abortion or stillbirth					
No	2951(78.8)	2707(80.0)	3885(83.1)	3822(85.0)	4117(83.2)
Yes	792(21.2)	669(19.8)	787(16.8)	672(15.0)	831(16.8)
Birth by caesarean section					
No	3526(94.3)	3031(89.6)	3833(82.0)	3405(75.8)	3288(66.5)
Yes	213(5.7)	351(10.4)	839(18.0)	1088(24.2)	1655(33.5)
Number of antenatal care visits					
Zero	1517(40.5)	1209(35.7)	1424(30.5)	964(21.5)	405(8.2)
1 visit	598(16.0)	530(15.7)	654(14.0)	731(16.3)	620(12.5)
2–3 visits	918(24.5)	832(24.6)	1271(27.2)	1358(30.2)	1534(31.0)
4 or more visits	708(18.9)	812(24.0)	1323(28.3)	1440(32.0)	2389(48.3)

^a^Rangpur was listed as a division in 2011. ^b^ Mymensingh was listed as a division in 2017.

**Figure 2:**
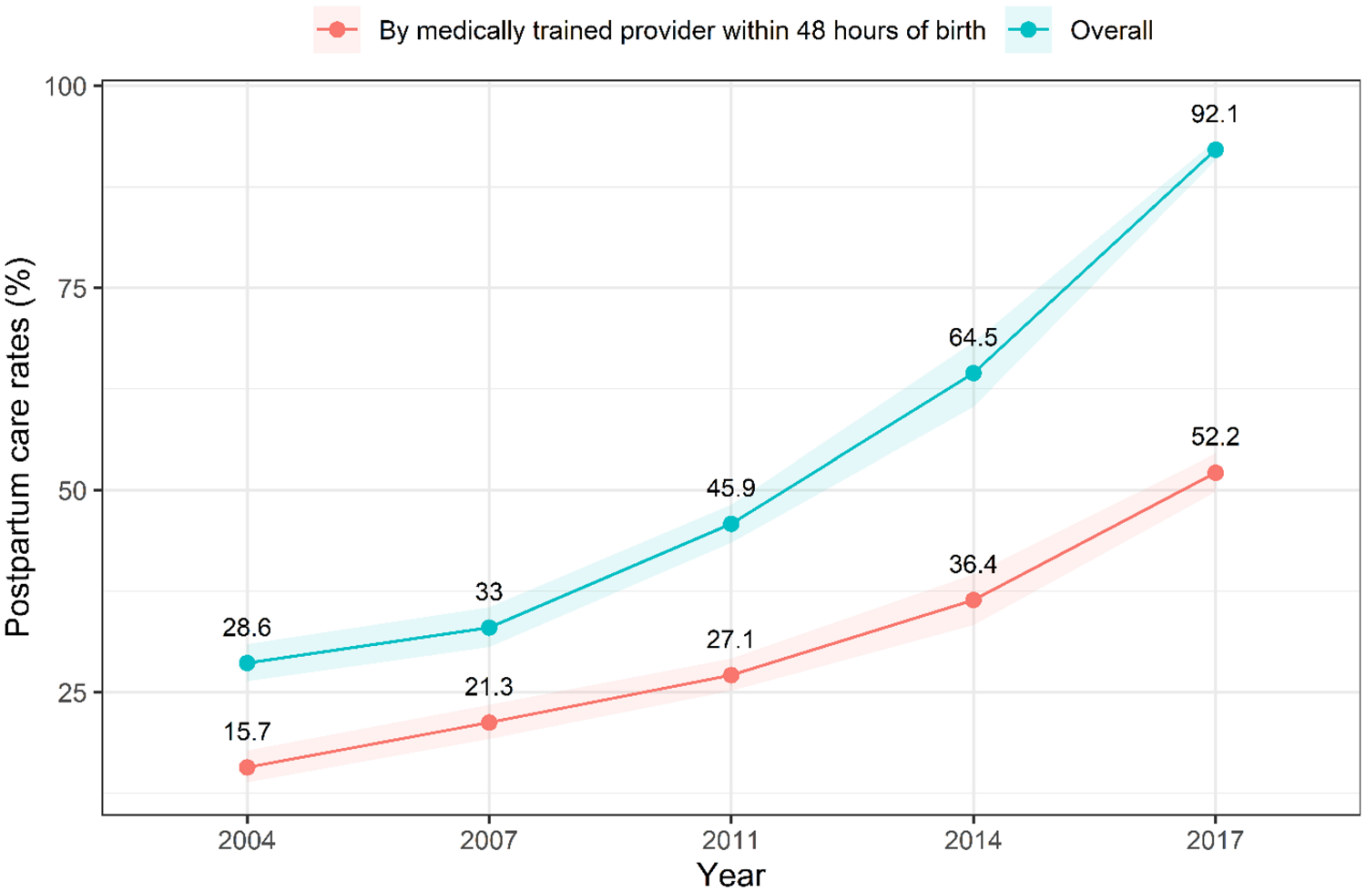
Trends in postnatal care rates in Bangladesh from 2004–2017.

Similarly, minimal changes in the proportion of women who had given birth at different age groups were observed between 2004 to 2017. The number of women who had a miscarriage, abortion or stillbirth and birth order 4 or more declined moderately between 2004 and 2017, however, the proportion of birth by caesarean section and 4 or more ANC visits increased substantially over time. Figure 2 demonstrates the trends in the PNC rate over time. The overall PNC rate increased from 28.8% in 2004 to 92.1% in 2017, while the rate of PNC provided by a ‘medically trained provider’ within 2 days of birth increased from 18.6% in 2004 to 52.2% in 2017.

**Figure 3:**
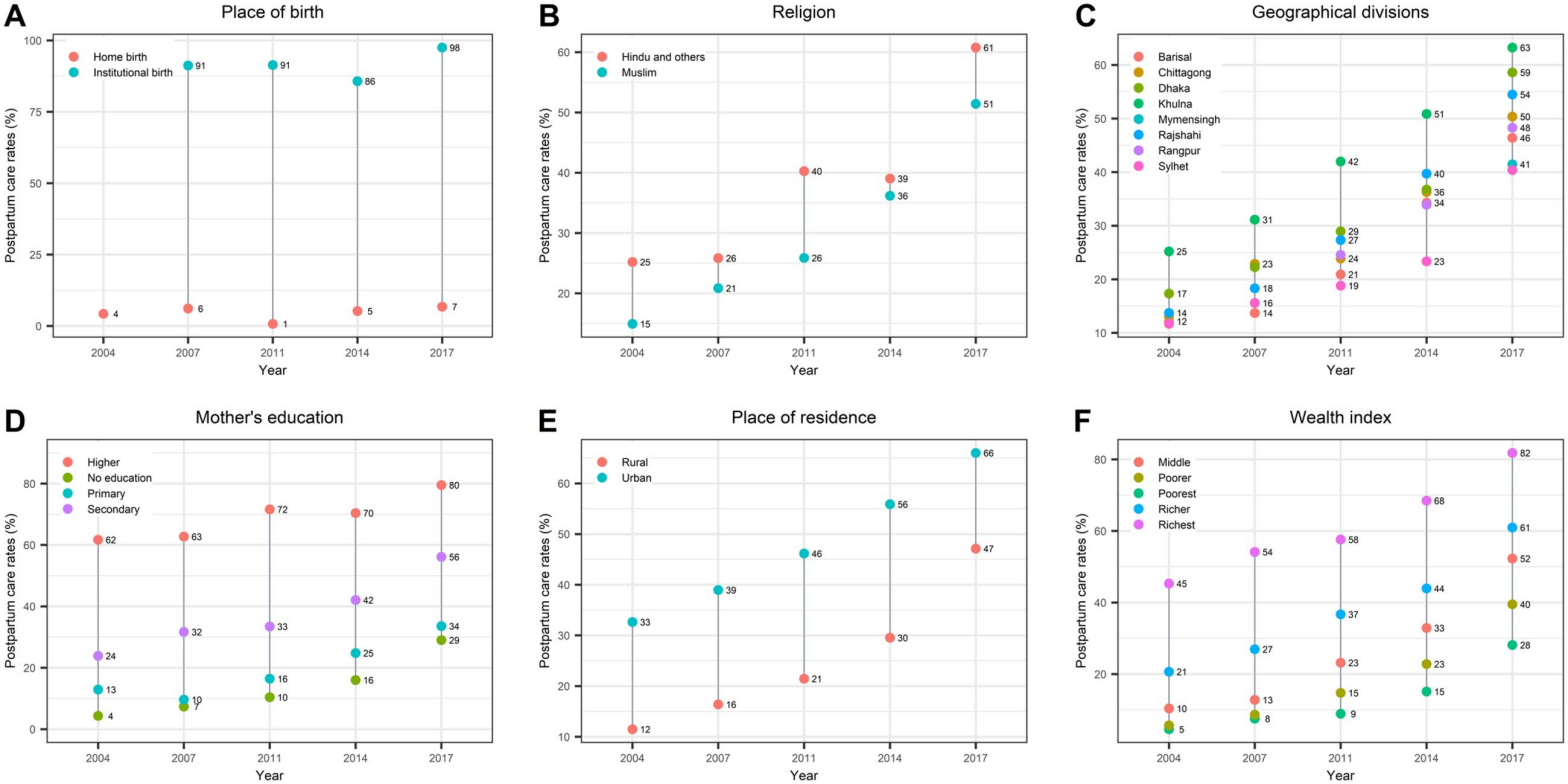
Trends in PNC rates by ‘medically trained provider’ within 2 days of birth by selected socio-demographic variables from 2004–2017.

Table 2 reports the percentage of women who received PNC by ‘medically trained provider’ within 48 h of birth across six socio-demographic characteristics for each survey. PNC rate was consistently low for women giving birth at home, living in rural areas, women who were Muslim, women with no formal education and women belonging to the poorest wealth quintile (Fig. 3). Among the eight administrative divisions, the PNC rate was consistently highest in the Khulna division (located in the south-west region of Bangladesh) in all surveys and lowest in Sylhet division (located in the north-eastern region of Bangladesh), in most surveys except 2007 (Table 2, Fig. 3C).

**Table 2:** Trends in PNC rate by ‘medically trained provider’ within 2 days of birth for women who has given birth three years preceding the survey by socio-demographic characteristics.

	Postnatal care rate for mothers				
	2004	2007	2011	2014	2017
	Rate (95% CI)	Rate (95% CI)	Rate (95% CI)	Rate (95% CI)	Rate (95% CI)
PNC rate	28.6 (26.4, 31.0)	33.0 (30.6, 35.5)	45.9 (43.5, 48.2)	64.5 (60.4, 68.4)	92.1 (90.8, 93.3)
PNC by medically trained provider within 2 days	15.7 (13.9, 17.8)	21.3 (19.2, 23.5)	27.1 (25.2, 29.2)	36.4 (33.4, 39.6)	52.2 (49.8, 54.5)
Place of birtha					
Institutional birth		91.2 (88.2, 93.6)	91.4 (89.5, 93.0)	85.8 (81.7, 89.0)	97.5 (96.7, 98.2)
Home birth	4.3 (3.5, 5.2)	6.1 (5.1, 7.3)	0.8 (0.5, 1.2)	5.2 (4.2, 6.5)	6.8 (5.6, 8.2)
Residence					
Urban	32.7 (27.6, 38.1)	39.0 (34.4, 43.8)	46.2 (41.9, 50.5)	55.9 (51.2, 60.5)	66.0 (62.5, 69.4)
Rural	11.5 (9.9, 13.4)	16.4 (14.2, 18.8)	21.5 (19.4, 23.7)	29.5 (26.3, 33.1)	47.1 (44.3, 50.0)
Divisionsb					
Barisal	12.2 (8.2, 17.6)	13.7 (9.7, 19.0)	20.9 (16.3, 26.5)	34.3 (23.9, 46.4)	46.4 (39.8, 53.1)
Chittagong	13.0 (9.2, 18.1)	22.9 (17.5, 29.3)	23.9 (19.9, 28.4)	36.3 (30.1, 42.3)	50.4 (44.4, 56.3)
Dhaka	17.3 (13.6, 21.8)	22.3 (18.4, 26.7)	29.0 (24.8, 33.4)	36.8 (30.2, 43.9)	58.6 (53.0, 64.1)
Khulna	25.2 (20.4, 30.9)	31.1 (25.8, 37.0)	42.0 (37.0, 47.2)	50.9 (44.6, 57.1)	63.3 (58.0, 68.2)
Mymensingh	/	/	/	/	41.5 (36.4, 46.7)
Rajshahi	13.7 (10.2, 18.3)	18.3 (15.0, 22.2)	27.3 (22.0, 33.4)	39.7 (34.0, 45.8)	54.5 (48.9, 59.9)
Rangpur	/	/	24.6 (20.3, 29.5)	33.9 (29.3, 38.9)	48.3 (40.9, 55.8)
Sylhet	11.7 (8.6, 15.7)	15.6 (11.8, 20.3)	18.8 (14.8, 23.7)	23.4 (17.6, 30.4)	40.4 (33.4, 47.8)
Religion					
Islam	15.0 (13.0, 17.1)	20.9 (18.8, 23.1)	25.9 (23.9, 28.0)	36.2 (33.1, 39.4)	51.4 (49.0, 53.8)
Hindu and others	25.2 (19.5, 31.9)	25.8 (17.7, 36.1)	40.3 (34.1, 46.7)	39.0 (27.5, 52.0)	60.8 (52.7, 68.3)
Mother’s education					
No education	4.3 (3.3, 5.7)	7.4 (5.6, 9.6)	10.4 (8.0, 13.4)	16.0(12.4, 20.4)	29.0 (23.7, 35.0)
Primary	12.9 (10.7, 15.4)	9.6 (7.8, 11.8)	16.4 (14.0, 19.2)	24.8 (21.3, 28.7)	33.6 (30.0, 37.3)
Secondary	23.9 (20.7, 27.4)	31.7 (28.3, 35.3)	33.4 (30.9, 36.1)	42.0 (38.4, 45.8)	56.1 (53.5, 58.7)
Higher	61.7 (53.1, 69.6)	62.7 (55.8, 69.2)	71.6 (65.9, 76.7)	70.4 (65.4, 74.9)	79.5 (76.8, 82.5)
Wealth index					
Poorest	4.6 (3.2, 6.6)	7.6 (5.5, 10.3)	8.9 (7.0, 11.2)	15.1 (12.0, 18.8)	28.1 (24.7, 31.9)
Poorer	5.7 (4.1, 7.9)	8.6 (6.5, 11.5)	14.8 (12.2, 17.8)	22.8 (19.5. 26.5)	39.5 (35.7, 43.5)
Middle	10.4 (8.2, 13.0)	12.8 (10.0, 16.2)	23.2 (20.3, 26.4)	32.9 (27.4, 38.9)	52.3 (48.1, 56.4)
Richer	20.7 (17.3, 24.6)	27.0 (22.3, 32.2)	36.7 (33.2, 40.4)	43.9 (38.9, 49.2)	61.0 (57.3, 64.6)
Richest	45.3 (40.7, 50.0)	54.1 (49.2, 59.0)	57.6 (53.2, 61.9)	68.5 (64.6, 72.1)	81.8 (79.0, 84.4)

^a^ in 2004 PNC questions were administered only for home birth ^b^ Rangpur and Mymensingh were listed as a division in 2011 and 2017 respectively.

Both the absolute and relative socio-demographic inequalities in accessing PNC by ‘medically trained provider’ within 48 h of birth is reported for each round in Table 3. Inequality between subgroups for place of birth was only measured from 2007 onwards (i.e., no data were available for 2004) (Table 2). Most of the exposure variables exhibited no definite upward or downward trend in absolute and relative inequalities and remain relatively persistent over time. However, some of the variables consistently showed a very high magnitude of inequality. For example, the highest level of inequality was observed in place of birth (institutional birth—home birth) in each survey—over 80% in terms of risk difference (absolute inequality) and over 14.0 in terms of relative risk (relative inequality). The absolute inequality for education level and wealth index remained consistently higher between time points (Table 3,), however, the relative inequality declined significantly for both the variables- RR 14.15 (95% CI 9.97, 18.42) in 2004 to RR 2.74 (95% CI 2.20, 3.28) in 2017 for education level and RR 9.81 (95% CI 6.25, 13.37) in 2004 to RR 2.90 (95% CI 2.52, 3.29) in 2017 for wealth index (Table 3).

**Table 3:** Trends in socio-demographic inequalities in PNC utilization by ‘medically trained provider’ within 2 days of birth for women who has given birth three years preceding the survey in Bangladesh from 2004–2017.

	Survey year				
	2004	2007	2011	2014	2017
	N = 3743	N = 3383	N = 4672	N = 4494	N = 4948
Place of birth *(Institutional birth—Home birth)*					
Absolute inequality RD^a^ % (95% CI)	/	85.1 (82.2, 88.0)	90.6 (88.8, 92.4)	80.5 (76.9, 84.1)	90.7 (89.3, 92.2)
Relative inequality RR^b^ (95% CI)	/	14.9 (12.2, 17.6)	117.6 (64.9, 170.3)	16.4 (12.8, 19.9)	14.4 (11.6, 17.1)
Residence *(Urban–Rural)*					
Absolute inequality RD % (95% CI)	21.1 (15.7, 26.6)	22.6 (17.4, 27.8)	24.7(19.8, 29.6)	26.3 (20.6, 32.1)	18.9 (14.4, 23.4)
Relative inequality RR (95% CI)	2.8 (2.2, 3.5)	2.4 (1.9, 2.8)	2.2 (1.9, 2.5)	1.9 (1.6, 2.2)	1.4 (1.3, 1.5)
Divisions *(Khulna-Sylhet)*					
Absolute inequality RD % (95% CI)	13.6 (7.5, 19.7)	15.6 (8.7, 22.4)	23.2 (16.3, 30.0)	27.5 (18.5, 36.5)	22.9 (14.2, 31.6)
Relative inequality RR (95% CI)	2.2 (1.4, 2.9)	2.0 (1.4, 2.6)	2.2 (1.6, 2.8)	2.2 (1.5, 2.8)	1.6 (1.3, 1.9)
Religion *(Hindu and Others-Islam)*					
Absolute inequality RD % (95% CI)	10.3 (4.0, 16.5)	5.0 (-4.6,14.6)	14.4 (7.7, 21.0)	2.9 (-9.9, 15.6)	9.3 (1.5, 17.2)
Relative inequality RR (95% CI)	1.7 (1.2, 2.1)	1.2 (0.8, 1.7)	1.6 (1.3, 1.8)	1.1 (0.7, 1.4)	1.2 (1.0, 1.3)
Mother’s education *(Higher-no education)*					
Absolute inequality RD % (95% CI)	57.3 (49.0, 65.6)	55.3 (48.1, 62.7)	61.2 (55.1, 67.3)	54.4 (48.5, 60.4)	50.5 (44.2, 56.8)
Relative inequality RR (95% CI)	14.2 (10.0, 18.4)	8.5 (6.0, 11.0)	6.9 (5.1, 8,7)	4.4 (3.3, 5.5)	2.7 (2.2, 3.3)
Wealth index *(Richest-poorest)*					
Absolute inequality RD % (95% CI)	40.7 (35.9, 45.5)	46.6 (41.0, 52.1)	48.7 (43.9, 53.5)	53.4 (48.4, 58.4)	53.7 (49.2, 58.2)
Relative inequality RR (95% CI)	9.8 (6.3, 13.4)	7.2 (4.8, 9.5)	6.5 (4.9, 8.0)	4.5 (3.5, 5.6)	2.9 (2.5, 3.3)

^a^Risk Difference ^b^Risk Ratio.

Table 4 shows the results of both unadjusted and adjusted analyses of the association of socio-demographic and maternal characteristics with PNC rate by a ‘medically trained provider’ within 2 days of birth in 2017. Among all the adjusted variables, women’s place of birth showed significant and remarkable association with PNC rate—women giving birth in a health facility were nearly 13 times more likely to receive PNC (aPR: 12.88 [95% CI 11.07, 14.97]) compared to women given birth at home. In two administrative divisions (Barisal and Chittagong), wealth index and number of ANC visits also showed a significant association, but the magnitude of the association was modest. For example, women who received at least 4 ANC visits had 28% higher rates (aPR: 1.28 [95% CI 1.10, 1.48]) of PNC compared to women who had no visit during their last childbirth (Table 4). Although place of residence, religion, mother’s education, twin pregnancy, miscarriage/abortion/stillbirth, and birth by caesarean section were found to be significantly associated with PNC care rates, none of them was significant in the adjusted analysis.

**Table 4:** Modified log Poisson regression analysis showing unadjusted and adjusted prevalence ratio of PNC by a ‘medically trained provider’ with 2 days of birth in relation to socio-demographic and maternal characteristics in 2017.

	Unadjusted PR (95%CI) (n = 4943)	Adjusted PR (95%CI) (n = 4943)
Place of birth (ref: Home birth)		
Institutional birth	13.90 (12.03,16.05)	12.88 (11.07,14.97) ***
Residence (ref: Rural)		
Urban	1.42 (1.35,1.49)	1.01 (0.98,1.03)
Division (ref: Sylhet)		
Barisal	1.09 (0.97,1.23)	1.14 (1.07,1.21)***
Chittagong	1.05 (1.03,1.27)	1.05 (1.01,1.10) *
Dhaka	1.35 (1.21,1.49)	1.02 (0.98,1.05)
Khulna	1.46 (1.32,1.62)	1.04 (0.99,1.08)
Mymensingh	1.00 (0.89,1.14)	1.04 (0.99,1.09)
Rajshahi	1.26 (1.13,1.41)	1.03 (0.99,1.07)
Rangpur	1.16 (1.03,1.30)	1.01 (0.97, 1.05)
Religion (ref: Islam)		
Hindu and others	1.18 (1.09,1.28)	0.98 (0.95, 1.01)
Mother’s education (ref: No education)		
Primary	1.23 (1.02,1.51)	0.99 (0.90, 1.09)
Secondary	2.06 (1.71,2.48)	1.04 (0.95,1.15)
Higher	2.98(2.47,3.58)	1.02 (0.93, 1.13)
Wealth index (ref: poorest)		
Poorer	1.42(1.26,1.61)	1.05 (1.00, 1.11)
Middle	1.92(1.71,2.15)	1.10 (1.04, 1.16) ***
Richer	2.22(1.99,2.47)	1.08 (1.03, 1.14) **
Richest	2.95(2.68,3.27)	1.12 (1.06, 1.18) ***
Mother’s age at last birth (ref: < 20)		
20–34	1.01 (0.95,1.07)	1.00 (0.97, 1.03)
35–49	0.97 (0.84,1.13)	1.04 (0.96, 1.01)
Sex of child (ref: Female)		
Male	1.03 (0.98,1.08)	0.98 (0.96, 1.01)
Child is twin (ref: singleton)		
Multiple	1.57 (1.38,1.79)	1.01 (0.96, 1.07)
Birth order (ref: 1)		
2–3	0.77 (0.73,0.81)	0.97 (0.94, 0.99) *
4–5	0.52 (0.45,0.59)	0.95 (0.89, 1.01)
6 +	0.30 (0.20, 0.44)	1.00 (0.75, 1.33)
Miscarriage/abortion/stillbirth (ref: No)		
Yes	1.10 (1.03, 1.17)	1.01 (0.98, 1.04)
Caesarean section (re: No)		
Yes	3.33 (3.16, 3.51)	1.00 (0.99, 1.02)
Number of antenatal care visits (ref: no visit)		
1 visit	2.01 (1.52, 2.64)	1.18 (1.02, 1.38) *
2–3 visits	3.53 (2.75, 4.52)	1.27 (1.10, 1.47) **
At least 4 visits	4.96 (3.89, 6.34)	1.28 (1.10, 1.48) ***

Exponentiated coefficients; 95% confidence intervals in brackets * p < 0.05, ** p < 0.01, *** p < 0.001.

## Discussion

Bangladesh has made remarkable progress in improving overall maternal health. Women’s receipt of PNC by a medically trained provider within the critical 48 h after birth has increased—from 16% in 2004 to 52% in 2017, and has also increased within all subgroups of six selected socio-demographic characteristics. However, significant inequalities between these sub-groups are evident. The single biggest factor affecting PNC use was whether a woman gave birth at home or in an institution. Other factors—living in rural rather than urban areas, living in certain geographical divisions, a woman’s education, and wealth index—were also significant.

Nearly half of women in Bangladesh given birth with the assistance of a traditional birth attendant, relatives or friends^38^. The presence of a skilled health provider is imperative to manage sudden risks and complications around the time of birth^45^. While births at a health facility have increased from 14% in 2004 to 49% in 2017, which is closer to the national target of 50%^5,39^, the rate of PNC by ‘medically trained provider’ within 2 days of birth for mothers given birth at home remained consistently low, indicating limited attention on PNC in primary level health services in Bangladesh.

In Bangladesh, more than 62% of people live in rural areas and almost two-thirds of births occur there^5^. This study demonstrated that the rate of PNC is persistently low in rural compared to urban areas. Distance to the nearest health facility center has been reported as one of the major obstacles for rural women in accessing services, which is evident in many studies^23,46^. A wide range of inequality across the administrative divisions in Bangladesh is observed across the surveys. The highest rate of PNC is observed in Khulna and the lowest in Sylhet in most surveys rounds, except in 2007. Notably, other studies have also observed significant variations across the administrative divisions for utilizing optimum maternal healthcare and Sylhet was found as the lowest performing division^47,48^. The possible reason might be the divergent availability and coverage of health facilities, including quality of services prevailing in the regions.

Persistent inequalities were observed among the richest and poorest mothers, and absolute inequality on the basis of wealth remained relatively unchanged between 2004 to 2017. Only 2.9% of the gross domestic product is allocated for health expenditure in Bangladesh, which resulted an explicitly high out-of-pocket health expenditure with 67%, one of the highest in the world^49^. Apparently, poor families and communities cannot afford the extra cost for health care, and this might be the major reason for such inequality. The study also revealed that the number of ANC visits is strongly associated with PNC, supporting evidence from other studies in Bangladesh, Nepal and Indonesia^23,50,51^, where antenatal visits were found to be a positive predictor of PNC utilization.

To the best of our knowledge, this is the first study that analysed the trends and inequalities of women’s use of PNC. We used Poisson regression models to identify factors associated with PNC, which provides robust estimates of the variance^40^. However, the cross-sectional nature of the survey may attribute to recall bias as the survey questionnaire is self-reporting. There is also a possibility that women may have incorrectly identified the care provider as a ‘medically trained provider’ that may result information bias^3^. BDHS survey questionnaires are also subject to reporting bias^52^. For example, prevailing social and cultural beliefs and stigmas may lead women to hide miscarriage, abortions, or stillbirths. These biases may either overestimate or underestimate the results of the study. However, considering the highly trained and efficient data collector recruited for the BDHS survey and one of the largest national standardised representative features of the BDHS, it is perceived that these biases may not have a large impact on the result of the study and are contemplated to be nominal. Another limitation is the BDHS data does not incorporate questions on the number of PNC, an important indicator to evaluate optimal care after childbirth.

In Bangladesh, the PNC rate for mothers has increased substantially over time; however, the increasing rate masks significant inequalities across key socio-demographic groups. The findings of this study call for greater health system strengthening to reduce these inequalities with particular focus on improving maternity services for women who receive no ANC, give birth unassisted at home, live in rural areas, live in regions with poor coverage of health services, and to women from low socio-economic groups. The findings further suggest the need for developing specific interventions and strategies considering these socio-economic and geographical inequalities. In addition, ensuring 4 + ANC visits and increasing institutional delivery would be crucial first steps in efforts to ensure timely PNC for all women. Special attention is needed for an effective monitoring and evaluation system for periodic evaluation of quality and coverage of PNC across the country. Considering the low coverage of maternal health service utilization in the country, the Government of Bangladesh has already provided a number of strategic directions in the Bangladesh National Strategy for Maternal Health 2019–2030^17^, including- prioritizing reducing existing inequalities in accessing and utilizing maternal health services, particularly for ANC, delivery, and PNC. Thus, the socio-demographic inequalities in PNC utilization identified in this study would be crucial to set priorities, develop national action plans and provide policy recommendations in maternal health to alleviate the progress towards reducing maternal and neonatal mortality and morbidity in Bangladesh.

## Methods

### Data sources

This study used data from the last five Bangladesh Demographic and Health Surveys (BDHS). BDHS is a cross-sectional, nationally representative surveillance survey that has been conducted periodically since 1993^32^. The survey is funded and administered by the Ministry of Health and Family Welfare, Bangladesh, and the United States Agency for International Development (USAID). The BDHS uses pre-designed questionnaires for households, women, men and communities. The women’s questionnaire collects information on several key reproductive, maternal and child health indicators.

The BDHS uses a two-stage, stratified cluster-sampling design, whereby primary sampling units (PSU) in both urban and rural areas across all administrative divisions are selected. Trained fieldworkers collect data through face-to-face household interviews using standardised questionnaires. Questions on postnatal care were first incorporated into BDHS in the 2004 survey, where data were collected only for women who gave birth at home (assuming that women given birth in any health facility will receive postnatal care, regardless of the mode of birth). From BDHS 2007 onwards the questionnaire was administered for all women giving birth preceding the survey, irrespective of the place of birth^33–37^. BDHS 2004 and 2007 were administered in six geographical divisions in Bangladesh^33,34^. BDHS 2011 onwards incorporated seven administrative divisions (including Rangpur)^35^ and the BDHS 2017 round was administered on eight administrative divisions after Mymensingh was declared a new division in 2015^38^. Data were publicly available from the Demographic and Health Survey website (https://dhsprogram.com) on request, therefore ethical approval was not required nor sought.

### Sample selection

Women aged 15–49 who had ever been married and had given birth in the five years preceding the 2004, 2007 and 2011 surveys were asked questions on postnatal care. In 2014 and 2017 these questions were directed at women who had given birth in the three years preceding the survey. For consistency, we have included only data from the five surveys on ever married women aged 15–49 who gave birth in the three years preceding the survey. The number of missing values were minimal for the study variables (i.e. only 1 or 2 participants per survey had missing data for the variables of interest). Hence, only data for participants with complete data for all variables were used.

### Postnatal care outcome

The primary outcome was the proportion of women who received postnatal care from a ‘medically trained provider’ within 2 days of birth. This outcome is derived from women’s responses to three survey questions: *‘Did anyone check on your health before discharge or after discharge/delivery at home?’, ‘Who checked on your health at that time?’* and *‘How many days or weeks after the delivery the first check take place?’.* A ‘medically trained provider’ could mean a range of health workers, whether a qualified doctor, nurse, midwife, paramedic, family welfare visitor, community skilled birth assistant or sub-assistant community medical officer^36^.

### Exposure variables

Six inequalities indicators were selected based on the review of the literature (including variables known to be associated with maternity service utilization in Bangladesh)^23,27,28,31^, and data available across all five surveys. These were place of birth (home birth/institutional birth), place of residence (urban/rural), geographical division, religion (Muslim/Hindu and others), mother’s education and wealth index. The DHS constructs the wealth index using data on household assets, combining both durable goods (land, bicycle, etc.) and basic amenities (household structure material, sanitation type and source of drinking water). Each asset was assigned a weight using principal component analysis (PCA) and standardized. Each household is assigned a score (summation of the total assets), which is applicable for all members residing in that household. The wealth index is divided into five quintiles of socioeconomic status: poorest, poorer, middle, richer and the richest^37^. PNC rates were measured for each available administrative division, for each survey. There were six administrative divisions in 2004 and 2007. Rangpur was added as a separate division, segregated from the Rajshahi division and later in 2017 Mymensingh was added as the eighth division, which was previously under the Dhaka division. Among the eight administrative divisions, Khulna had the highest rate of PNC in all surveys and Sylhet had the lowest in most surveys. Hence, to estimate inequalities in the PNC rate among divisions, we considered Khulna and Sylhet^39^.

### Factors associated with postnatal care coverage

Data from BDHS 2017 (the most recent data available) was used to identify the factors associated with low postnatal care coverage among women received care from a ‘medically trained provider’ within 2 days of birth. Along with the six socio-demographic indicators described above, additional individual level factors were also included as covariates in a Poisson regression model. Individual variables were identified through literature review and review of similar papers that used DHS data to explore associations related to PNC in other countries. We used: mother’s age at last birth, sex of the child, twin pregnancy, birth order, miscarriage, abortion or stillbirth (yes/no), birth by caesarean section (yes/no) and number of antenatal care (ANC) visits.

### Statistical analysis

Descriptive statistics (number and percentage) were used to report the characteristics of participants in each survey. To explore possible inequalities across socio-demographic indicators, we estimated the postnatal care rate with 95% CI and measured absolute inequality by estimating Rate Difference (RD) in PNC between subgroups (e.g., urban–rural) and relative inequality using Rate Ratio (RR) (e.g., urban/rural). For absolute inequality, a large RD refers to wider inequality between the subgroups. For relative inequality, RR > 1 indicates higher risk of the outcome in a subgroup compared to the reference group and RR < 1 indicates lower risk of the outcome in a selected subgroup compared to the reference group.

To determine the factors associated with the PNC utilization among mothers who received PNC from a ‘medically trained provider’ within 2 days of birth, a modified Poisson regression model was used to estimate the adjusted and unadjusted prevalence ratio with 95% CI^40,41^. The modified Poisson regression approach can estimate relative risks/prevalence ratios consistently and efficiently using a robust error variance^40^. Unlike binomial and Poisson regression models that are commonly used for estimating relative risks, this procedure is less prone to converging difficulties and provides more accurate estimates of the standard errors^40^. Survey weights were applied to all analyses for the accurate representation of the results^42^. Data were analysed using STATA 14.0 ^43^ with figures generated using R statistical software^44^.

## References

[1] World Health Organization (2019). Trends in maternal mortality 2000 to 2017: estimates by WHO, UNICEF, UNFPA, World Bank Group and the United Nations Population Division: executive summary.

[2] World Health Organization. WHO recommendations on Postnatal care of the mother and newborn. (2013). 24624481

[3] Fort AL (2012). Coverage of post-partum and post-natal care in Egypt in 2005–2008 and Bangladesh in 2004–2007: levels, trends and unmet need. Reprod. Health Matters.

[4] Technical Working Group, WHO. Postpartum care of the mother and newborn: a practical guide. *Birth* **26**, 255–258, doi:10.1046/j.1523-536x.1999.00255.x (1999). 10655832

[5] ACOG Committee Opinion No (2018). 736: Optimizing Postpartum Care. Obstet. Gynecol..

[6] Lawn JE (2014). Every Newborn: progress, priorities, and potential beyond survival. Lancet.

[7] Marmot M (2005). Social determinants of health inequalities. Lancet.

[8] Adeyanju O, Tubeuf S, Ensor T (2017). Socio-economic inequalities in access to maternal and child healthcare in Nigeria: changes over time and decomposition analysis. Health Policy Plan..

[9] Goli S, Nawal D, Rammohan A, Sekher TV, Singh D (2018). Decomposing the socioeconomic inequality in utilization of maternal health care services in selected countries of South Asia and Sub-Saharan Africa. J. Biosoc. Sci..

[10] John, B. Trends in Maternal Mortality: 1990 to 2015 WHO, UNICEF, UNFPA, World Bank Group, and United Nations Population Division. 726 (2016).

[11] Langlois ÉV (2015). Inequities in postnatal care in low- and middle-income countries: a systematic review and meta-analysis. Bull. World Health Org..

[12] The United Nations. *The Sustainable Development Goals Report 2019*, https://unstats.un.org/sdgs/report/2020/The-Sustainable-Development-Goals-Report-2020.pdf (2020).

[13] The World Bank. *World Development Indicators database. Bangladesh Country Profile*, https://databank.worldbank.org/views/reports/reportwidget.aspx?Report_Name=CountryProfile&Id=b450fd57&tbar=y&dd=y&inf=n&zm=n&country=BGD (2020).

[14] Our world in data. *Which countries are most densely populated?*, https://ourworldindata.org/most-densely-populated-countries (2019).

[15] Index Mundi. *Bangladesh Demographics Profile*, https://www.indexmundi.com/bangladesh/demographics_profile.html (2020).

[16] Obstetrical and Gynaecological Society of Bangladesh (OGSB), Ministry of Health and Family welfare (MOHFW), Directorate General of Health Services (DGHS), Directorate General of Family Planning (DGFP). Standard Clinical Management Protocols and Flowcharts on Emergency Obstetric and Neonatal Care. (2019).

[17] Ministry of Health and Family Welfare (MOHFW). Bangladesh National Strategy for Maternal Health 2019–2030. (Directorate General of Health Services (DGHS), Ministry of Health and Family Welfare (MOHFW), Dhaka, Bangladesh, 2019).

[18] Shahjahan M, Chowdhury HA, Al-Hadhrami AY, Harun GD (2017). Antenatal and postnatal care practices among mothers in rural Bangladesh: A community based cross-sectional study. Midwifery.

[19] Keya KT, Rob U, Rahman M, Bajracharya A, Bellows B (2014). Distance, transportation cost, and mode of transport in the utilization of facility-based maternity services: evidence from rural Bangladesh. Int. Quart. Comm. Health Educ..

[20] Syed U, Asiruddin S, Helal MS, Mannan II, Murray J (2006). Immediate and early postnatal care for mothers and newborns in rural Bangladesh. J. Health Popul. Nutr..

[21] Choudhury N (2012). Beliefs and practices during pregnancy and childbirth in urban slums of Dhaka, Bangladesh. BMC Publ. Health.

[22] Amin R, Shah NM, Becker S (2010). Socioeconomic factors differentiating maternal and child health-seeking behavior in rural Bangladesh: a cross-sectional analysis. Int. J. Equity Health.

[23] Rahman MM, Haque SE, Zahan MS (2011). Factors affecting the utilisation of postpartum care among young mothers in Bangladesh. Health Soc. Care Comm..

[24] Chakraborty N, Islam MA, Chowdhury RI, Bari W (2002). Utilisation of postnatal care in Bangladesh: evidence from a longitudinal study. Health Soc. Care Commun..

[25] Goldenberg T, Stephenson R (2017). A Deviance approach to understanding use of maternal health care services in Bangladesh. Int. Perspect. Sex. Reprod. Health.

[26] Khan MN, Kumar P, Rahman MM, Islam Mondal MN, Islam MM (2020). Inequalities in utilization of maternal reproductive health care services in Urban Bangladesh: a population-based study. SAGE Open.

[27] Pulok MH, Uddin J, Enemark U, Hossin MZ (2018). Socioeconomic inequality in maternal healthcare: an analysis of regional variation in Bangladesh. Health Place.

[28] Mahabub-Ul-Anwar M, Rob U, Talukder MN (2006). Inequalities in maternal health care utilization in rural Bangladesh. Int. Quart. Comm. Health Edu..

[29] Hajizadeh M, Alam N, Nandi A (2014). Social inequalities in the utilization of maternal care in Bangladesh: have they widened or narrowed in recent years?. Int. J. Equity Health.

[30] Anwar, A., Killewo, J., Chowdhury, M. & Dasgupta, S. Bangladesh: inequalities in utilization of maternal health care services-evidence from Matlab. *Washington DC World Bank* (2004).

[31] Anwar I (2008). Inequity in maternal health-care services: evidence from home-based skilled-birth-attendant programmes in Bangladesh. Bull. World Health Org..

[32] The DHS Program. *Data*, https://dhsprogram.com/data/

[33] National Institute of Population Research and Training (NIPORT), Mitra and Associates, and ORC Macro. Bangladesh Demographic and Health Survey (2004). (National Institute of Population Research and Training, Mitra and Associates, and ORC Macro.

[34] National Institute of Population Research and Training (NIPORT), Mitra and Associates, and Macro International. Bangladesh Demographic and Health Survey (2007). (National Institute of Population Research and Training, Mitra and Associates, and ORC Macro.

[35] National Institute of Population Research and Training (NIPORT), Mitra and Associates/Bangladesh, and ICF International. Bangladesh Demographic and Health Survey (2011). (NIPORT, Mitra and Associates, and ICF International.

[36] National Institute of Population Research and Training (NIPORT), Mitra and Associates, and ICF International. Bangladesh Demographic and Health Survey (2014). (NIPORT, Mitra and Associates, and ICF International.

[37] The DHS Program. *Wealth-Index-Construction*, https://www.dhsprogram.com/topics/wealth-index/Wealth-Index-Construction.cfm

[38] National Institute of Population Research and Training (NIPORT), and ICF. Bangladesh Demographic and Health Survey 2017–2018. (NIPORT and ICF, Dhaka, Bangladesh, and Rockville, Maryland, USA, 2020).

[39] Anwar I, Nababan HY, Mostari S, Rahman A, Khan JAM (2015). Trends and inequities in use of maternal health care services in Bangladesh, 1991–2011. PLoS ONE.

[40] Zou G (2004). A modified poisson regression approach to prospective studies with binary data. Am. J. Epidemiol..

[41] Yisma, E., Smithers, L. G., Lynch, J. W. & Mol, B. W. Cesarean section in Ethiopia: prevalence and sociodemographic characteristics. *J. Mater. Fetal Neonatal Med. Off. J. Eur. Assoc. Perinat. Med. Federat. Asia Ocean. Perinatal Soc. Int. Soc. Perinatal Obstet* **32**, 1130–1135, doi:10.1080/14767058.2017.1401606 (2019). 10.1080/14767058.2017.1401606 29103331

[42] The DHS Program. *Using Datasets for Analysis*, https://www.dhsprogram.com/data/Using-Datasets-for-Analysis.cfm

[43] Stata Statistical Software (2015). Release 14.

[44] R: A language and environment for statistical computing (R Foundation for Statistical Computing, Vienna, Austria, 2020).

[45] Akanda M, Salam A (2012). Demand for institutional delivery in Bangladesh: an application of household production function. Dhaka Univ. J. Sci..

[46] Bhatia, J. C. & Cleland, J. Determinants of maternal care in a region of South India. *Health Trans. Rev.*, 127–142 (1995).

[47] Islam MM, Masud MS (2018). Determinants of frequency and contents of antenatal care visits in Bangladesh: assessing the extent of compliance with the WHO recommendations. PLoS ONE.

[48] Islam MM, Masud MS (2018). Health care seeking behaviour during pregnancy, delivery and the postnatal period in Bangladesh: assessing the compliance with WHO recommendations. Midwifery.

[49] Islam MA, Akhter S, Islam M (2018). Health financing in Bangladesh: why changes in public financial management rules will be important. Health Syst. Reform.

[50] Khanal V, Adhikari M, Karkee R, Gavidia T (2014). Factors associated with the utilisation of postnatal care services among the mothers of Nepal: analysis of Nepal demographic and health survey 2011. BMC Womens Health.

[51] Titaley CR, Dibley MJ, Roberts CL (2009). Factors associated with non-utilisation of postnatal care services in Indonesia. J. Epidemiol. Comm. Health.

[52] Rahman MM (2018). Determinants of caesarean section in Bangladesh: cross-sectional analysis of Bangladesh Demographic and Health Survey 2014 Data. PLoS ONE.

